# A Prospective Study of Vitamin and Mineral Supplement Use and the Risk of Upper Gastrointestinal Cancers

**DOI:** 10.1371/journal.pone.0088774

**Published:** 2014-02-18

**Authors:** Sonja P. Dawsey, Albert Hollenbeck, Arthur Schatzkin, Christian C. Abnet

**Affiliations:** 1 Nutritional Epidemiology Branch, Division of Cancer Epidemiology and Genetics, National Cancer Institute, Rockville, Maryland, United States of America; 2 AARP, Washington, DC, United States of America; Sookmyung Women’s University, Republic of Korea

## Abstract

We examined the association of use of multivitamins or single vitamin/mineral supplements with risk of four upper gastrointestinal cancers in the NIH-AARP Diet and Health Study cohort with 11 years of follow-up. After exclusions, 490,593 persons were included in our analytic cohort and 1780 upper gastrointestinal cancers were accrued. Hazard ratios (HRs) and 95% confidence intervals (CIs) were calculated using Cox models with adjustment for potential confounders. We observed no significant associations between multivitamin use and risk for the four cancer outcomes in crude or adjusted models. Among individual vitamin or mineral supplements, use of iron supplements was associated with significantly lower risk of esophageal adenocarcinoma (HR = 0.68, 95% CI = 0.49 to 0.94) and a significantly increased risk of gastric noncardia adenocarcinoma (HR = 1.59, 95% CI = 1.24 to 2.05). For gastric noncardia adenocarcinoma, we saw associations with zinc use (HR = 1.28, 95% CI = 1.01 to 1.62) and vitamin C use (HR = 0.79 95% CI = 0.65 to 0.96). Calcium use, some of which was reported as antacids and used to treat reflux disease, was associated with higher risk of esophageal adenocarcinoma (HR = 1.27, 95% CI = 1.06 to 1.52) and gastric cardia adenocarcinoma (HR = 1.27, 95% CI = 1.03 to 1.56) cancers. We saw no evidence that multivitamin use was associated with reduced risk of four highly fatal upper gastrointestinal cancers, but there were some differences in risk with reported use of individual supplements.

## Introduction

Fifty-three percent of US adults report using vitamin supplements [1], including about 35% that use multivitamin supplements [2]. The purported health benefits of these agents include potentially lowering cancer risk, but few benefits of any sort have been proven in clinical trials. Any benefits in preventing cancer may be organ specific and may depend on the nutritional status of those taking the supplements.

Esophageal cancer and gastric cancer are the 6th and 2nd most common causes of cancer death worldwide [3]. Of the two histological types of esophageal cancer, esophageal squamous cell carcinoma (ESCC) is more frequent worldwide, but a recent rise in esophageal adenocarcinoma incidence rates now makes it the more frequent type in Western countries [3]. Adenocarcinomas make up the majority of gastric cancers, but because of apparent etiologic difference they are often analyzed separately by tumor location within the stomach as gastric cardia adenocarcinoma and gastric noncardia adenocarcinoma. In developed countries, the incidence of gastric cardia adenocarcinoma may have risen in the past few decades, accompanying the rise in esophageal adenocarcinoma [4], but this is not clear [5]. These cancers are uncommon in the US and there are no standard screening programs. Patients with these cancers are typically diagnosed at a late stage leading to poor survival, making preventive strategies particularly important.

Several studies have investigated the relationship between vitamin supplementation and risk of upper gastrointestinal cancers. The General Population Nutrition Intervention Trial in Linxian, China (a randomized clinical trial) showed a protective effect against gastric cancer when participants were supplemented with a combination of selenium, β-carotene, and α-tocopherol, and the benefit was still apparent 10 years after supplementation ended [6]. In contrast, the Dysplasia Trial conducted in the same population used a multivitamin supplement and showed no effect in subjects with preneoplastic lesions of the esophagus. Other studies, including a population based case-control study [7]–[8] and a cohort study [9] in Sweden and observational studies in the Woman’s Health Initiative [10] and the Cancer Prevention Study II Cohort [11] show null or varying borderline associations between multivitamin and other supplement use and esophageal or gastric cancer risk. Two meta-analyses evaluated the effect of antioxidants in prevention of gastrointestinal cancers [12] and the effect of antioxidants as a primary and secondary method of mortality prevention in randomized supplement trials [13]. These studies concluded that there appeared to be little evidence that antioxidant supplements would reduce the risk of these cancers.

Overall, the available data on the relationship between vitamin and mineral supplementation and upper gastrointestinal cancer risk are sparse and inconclusive. The current study aims to examine the association between multivitamin and other supplement use in the prospective NIH-AARP Diet and Health Study cohort.

## Methods

### Study Population

The establishment and recruitment procedures of the NIH-AARP Diet and Health Study, a large prospective cohort study investigating the association between diet and other factors and risk of cancer, have been described in detail elsewhere [14]. Briefly, between 1995–1996 a questionnaire was mailed to 3.5 million AARP members (aged 50–71) in six U.S. states (California, Florida, Louisiana, New Jersey, North Carolina, and Pennsylvania) and two metropolitan areas (Atlanta, Georgia, and Detroit, Michigan). A total of 617,119 (18%) individuals returned the questionnaire and of those, 566,402 (92%) respondents filled out the survey in satisfactory detail and consented to be in the study. We excluded subjects with cancer at baseline (n = 51,234), proxy respondents (n = 15,760), those with calorie intake more than two inter-quartile ranges from the mean (n = 4,383), those who lacked complete information on supplement use (n = 4420), and those who died or were diagnosed with cancer on the first day of follow-up (n = 12). After these exclusions, our analytic cohort included 490,593 persons, of which 292,774 were men and 197,819 were women.

### Cohort Follow-up

As previously described [15], member addresses of the NIH-AARP cohort were updated annually by matching the cohort database to the National Change of Address database maintained by the U.S. Postal Service. Vital status was ascertained by linkage of the cohort to the Social Security Administration Death Master File of deaths in the U.S., cancer registry linkage, questionnaire responses, and responses to other mailings. Incident cases of cancer through December 31st 2006 were identified by probabilistic linkage between the NIH-AARP cohort membership and the state cancer registry databases of the eight states from which the cohort members were recruited, with the later addition of Arizona and Texas. All participating registries have been certified by the North American Association of Central Cancer Registries for meeting the highest standards of quality. All of these cancer registries are estimated to be 95% complete within two years of cancer incidence. For matching purposes, we had nearly complete data on first and last name, address history, gender, and date of birth. Social Security number was available for 85% of the participants. Cancer sites were identified by histologic code and anatomic site, as described previously [16] using the International Classification of Disease for Oncology, third edition. We classified tumors with site codes C15.0– C15.9 as esophageal cancers, site code C16.0 as gastric cardia tumors, and site codes C16.1– C16.9 as noncardia tumors. We treated C16.8 and C16.9 (gastric tumors without specific location information) as noncardia cancers. To assess the effect of this assignment we tested all subsequent results after excluding these cases and found no consequential differences.

### Exposure Assessment

We defined frequency of participant use of vitamin supplements as described in detail elsewhere [17] based on use over the 12 months before completion of the questionnaire. Briefly, supplement use was assessed for 3 types of multivitamins (“stress-tab type”, “therapeutic or theragran type”, “one-a-day-type”) and we collapsed these into use of any type of multivitamin to provide sufficient numbers of cases in each cell to provide stable estimates of association. Frequency of use was recorded as never, less than 1 time per week, 1–3 times per week, 4–6 times per week, or every day, but we collapsed these into four categories: none, less than 7 times per week, 7 times per week, or more than 7 times per week. Those classified as more that than 7 times per week were using at least two supplement types. For some analyses, this was collapsed further to any use versus none. Use of other single supplements (vitamin A, beta-carotene, vitamin C, vitamin E, calcium) was collected as the frequency of use for each supplement and this was collapsed to any versus none to provide sufficient numbers of cases in each cell to provide stable estimates of association. Finally, use of iron, zinc, selenium, and folic acid supplements was collected and analyzed as a dichotomous variable with exposure defined as use more than once per month.

### Statistical Analysis

Known or potential risk factors were investigated as possible confounders and were tabulated by supplement use. Hazard ratios (HRs) and 95% confidence intervals (CIs) were calculated using Cox proportional hazards regression with follow-up time as the underlying time metric. We included adjusting variables that changed beta values for vitamin use by ≥10% or had an independent association with cancer risk. We used a single model for all outcomes and adjusted estimates for age at cohort entry (continuous), sex, education (categorical), smoking status and intensity (categorical), alcohol use (continuous), fruit intake (continuous), vegetable intake (continuous), body mass index (BMI) (continuous), vigorous physical activity (categorical), usual physical activity during the day (categorical), and total energy intake (continuous). The adjusting covariates were modeled as presented in Table 1. If adjusting covariate values were missing for a subject, a dummy variable for missingness was used in the models and the frequency of missingness ranged from 1 to 4%. We tested for interactions with cancer risk using cross-product variables for any multivitamin use with sex or ever smoking separately. No significant deviations from proportionality were detected using variables for the interaction between any multivitamin use and time. We used SAS version 9.2 (SAS Institute, Inc., Cary, NC) for all analyses. We used two-sided tests and considered P-values <0.05 to be statistically significant.

**Table 1:** Cohort characteristics by frequency of multivitamin supplement use in the NIH-AARP Diet and Health Study cohort.

	Frequency of multivitamin use, no. of times per week			
	Never	1–6	7	>7
**Number of people, N (%)**	220385 (45)	59213 (12)	186166 (38)	24829 (5)
**Age, years, mean (s.d.)**	62.1 (5.4)	61.1 (5.4)	62.3 (5.3)	61.7 (5.4)
**Sex, male, N (%)**	141706 (64)	32555 (55)	104868 (56)	13645 (55)
**Education**				
**High school or less, N (%)**	69225 (31)	14424 (24)	49732 (27)	5863 (24)
**Post-high school training, N (%)**	71255 (32)	20290 (34)	62091 (33)	8488 (34)
**College graduate, N (%)**	40145 (18)	11543 (19)	35803 (19)	4829 (19)
**Post-graduate education, N (%)**	39760 (18)	12956 (22)	38540 (21)	5649 (23)
**Tobacco smoking**				
**Never, N (%)**	75475 (34)	22215 (38)	65499 (35)	9003 (36)
**Former <20 cigarettes per day, N (%)**	57601 (26)	15389 (26)	51010 (27)	6542 (26)
**Former ≥20 cigarettes per day, N (%)**	46619 (21)	10511 (18)	39612 (21)	5139 (21)
**Current <20 cigarettes per day, N (%)**	20300 (9)	5792 (10)	15377 (8)	2118 (9)
**Current ≥20 cigarettes per day, N (%)**	11549 (5)	3151 (5)	8047 (4)	1070 (4)
**Missing, N (%)**	8841 (4)	2155 (4)	6621 (4)	957 (4)
**Alcohol, drinks per day, mean (s.d.)**	1.0 (2.5)	0.9 (2.2)	0.9 (2.3)	0.9 (2.2)
**Fruit intake, servings per day, mean (s.d.)**	2.8 (2.4)	2.8 (2.3)	3.1 (2.5)	3.5 (2.8)
**Vegetable intake, servings per day, mean (s.d.)**	3.8 (2.4)	3.9 (2.4)	4.0 (2.5)	4.4 (2.9)
**Body mass index, kg m^−2^, mean (s.d.)**	27.4 (5.1)	27.0 (5.0)	26.8 (4.9)	27.0 (5.2)
**Calories, kcal/day, mean (s.d.)**	1850 (826)	1818 (797)	1816 (778)	1898 (853)
**Vigorous physical activity**				
**Never, N (%)**	14604 (7)	2397 (4)	8960 (5)	1101 (4)
**Rarely, N (%)**	33198 (15)	7830 (13)	22585 (12)	2860 (11)
**1–3 times per month, N (%)**	31056 (14)	9595 (16)	22672 (12)	3134 (13)
**1–2 times per week, N (%)**	46936 (21)	14859 (25)	38520 (21)	5164 (21)
**3–4 times per week, N (%)**	54900 (25)	16003 (27)	53106 (29)	7129 (29)
**≥5 times per week, N (%)**	39691 (18)	8529 (14)	40323 (22)	5441 (22)
**Activity throughout the day**				
**Sit during day, not much walking, N (%)**	22910 (10)	6284 (11)	17938 (10)	2783 (11)
**Sit much of the day, walk a fair amount, N (%)**	70120 (32)	19637 (33)	59931 (32)	8067 (32)
**Stand/walk a lot, no lifting, N (%)**	82567 (37)	21428 (36)	71450 (38)	9246 (37)
**Lift/carry light loads, N (%)**	37809 (17)	10269 (17)	31948 (17)	4070 (16)
**Heavy work, N (%)**	6979 (3)	1595 (3)	4899 (3)	663 (3)

## Results

After 11 years of follow up totaling 4,760,017 person-years we accrued 212 ESCC cases, 625 esophageal adenocarcinoma cases, 450 gastric cardia adenocarcinoma cases, and 493 gastric noncardia adenocarcinoma cases. Among this cohort, 220,385 (45%) reported no multivitamin use, 59,213 (12%) reported use less than 7 times per week, 186,166 (38%) reported using multivitamins 7 times per week, and 24,829 (5%) reported using them more than 7 times per week. Age, tobacco and alcohol use, BMI, physical activity throughout the day, and total energy intake were similar across different frequencies of supplement use (Table 1). Characteristics associated with more frequent multivitamin use included being female, higher levels of education, higher fruit and vegetable intake, and more frequent vigorous physical activity.

We observed no significant associations between multivitamin use and risk for any of the four cancer outcomes in crude models or models adjusted for age, sex, education, smoking, alcohol use, fruit and vegetable intake, BMI, physical activity, and total energy intake (Table 2).

**Table 2:** Crude and adjusted* hazard ratios (HR) and 95% confidence intervals (CI) for use of multivitamin supplements for four upper gastrointestinal cancers in the NIH-AARP Diet and Health Study cohort.

	Frequency of multivitamin use, no. of times per week				
	Never	1–6	7	>7	P_trend_
**Person-years**	2135548	579983	1804459	240027	
**ESCC**					
** No. cases**	96	24	81	11	
** Crude HR (95% CI)**	1.00 (ref)	0.92 (0.59–1.44)	1.00 (0.74–1.34)	1.02 (0.55–1.90)	0.98
** Multivariable HR (95% CI)**	1.00 (ref)	1.03 (0.66–1.62)	1.04 (0.77–1.41)	1.14 (0.61–2.13)	0.68
**EADC**					
** No. cases**	319	50	234	22	
** Crude HR (95% CI)**	1.00 (ref)	0.58 (0.43–0.78)	0.87 (0.73–1.03)	0.61 (0.40–0.95)	0.017
** Multivariable HR (95% CI)**	1.00 (ref)	0.73 (0.54–0.98)	1.02 (0.86–1.20)	0.75 (0.49–1.16)	0.64
**GCA**					
** No. cases**	209	43	176	22	
** Crude HR (95% CI)**	1.00 (ref)	0.76 (0.55–1.05)	1.00 (0.82–1.22)	0.94 (0.60–1.45)	0.88
** Multivariable HR (95% CI)**	1.00 (ref)	0.96 (0.69–1.34)	1.16 (0.94–1.42)	1.16 (0.74–1.80)	0.16
**GNCA**					
** No. cases**	245	53	179	16	
** Crude HR (95% CI)**	1.00 (ref)	0.80 (0.59–1.07)	0.87 (0.71–1.05)	0.58 (0.35–0.96)	0.028
** Multivariable HR (95% CI)**	1.00 (ref)	0.97 (0.72–1.30)	0.92 (0.76–1.12)	0.65 (0.39–1.08)	0.15

*Adjustments included age at cohort entry, sex, education, smoking status and intensity, alcohol use, fruit intake, vegetable intake, body mass index (BMI), vigorous physical activity, usual physical activity during the day, and total energy intake. ESCC = esophageal squamous cell carcinoma; EADC = esophageal adenocarcinoma; GCA = gastric cardia adenocarcinoma; GNCA = gastric noncardia adenocarcinoma.

**Figure 1:**
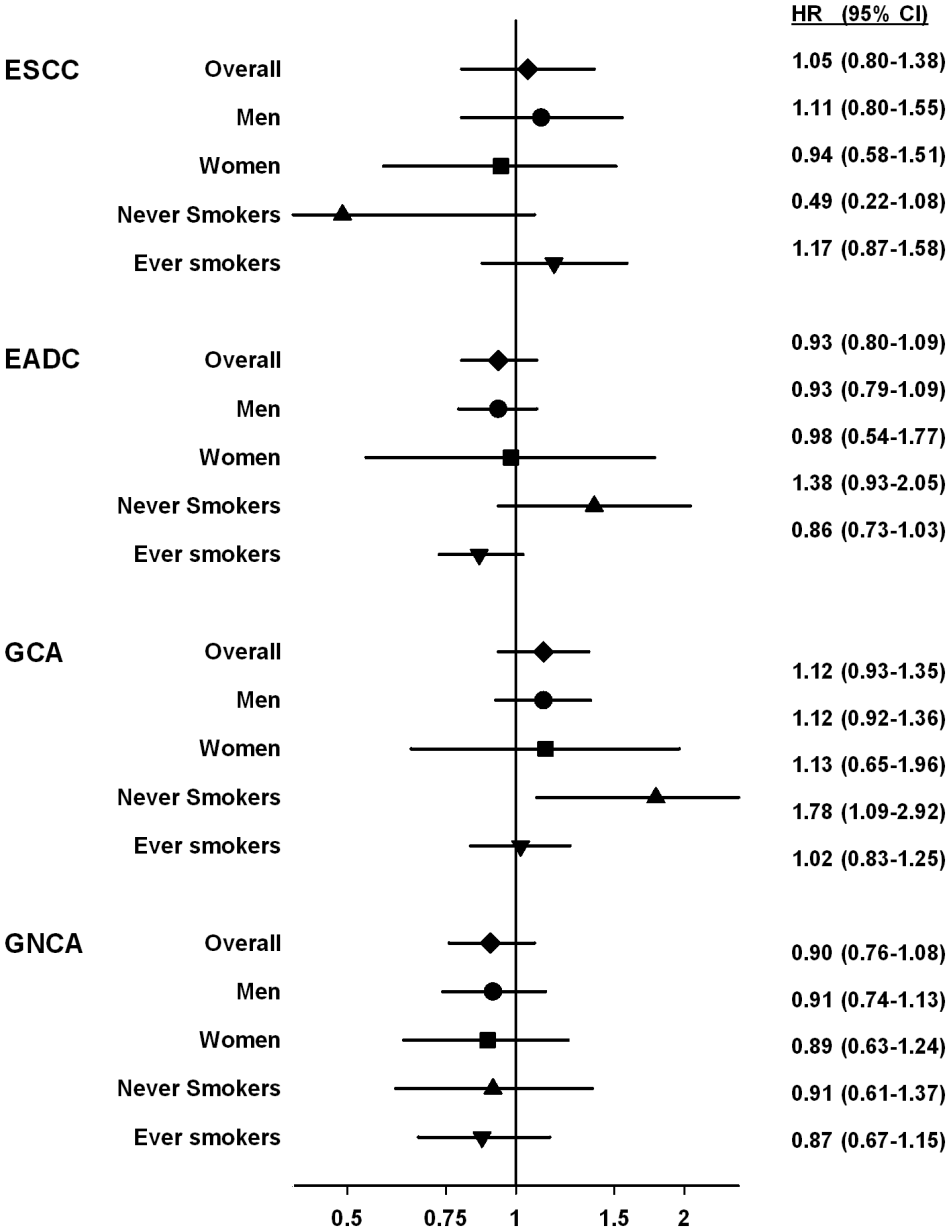
Adjusted hazard ratios (HR) and 95% confidence intervals (CI) for any use of multivitamin supplements for four upper gastrointestinal cancers stratified by sex or smoking in the NIH-AARP Diet and Health Study cohort. Of the eight tests, only smoking status for esophageal adenocarcinoma showed significant effect modification (P = 0.022), but the estimates in both strata have confidence intervals that include 1.

We tested for effect modification between any multivitamin use and sex or ever smoking for each of the four cancers (eight interaction tests). Figure 1 presents risk estimates for each of the four strata (men or women; never smoker or ever smoker). For seven of the eight stratifications there was no evidence of difference, but the interaction test for multivitamin use by smoking status was statistically significant for esophageal adenocarcinoma (P = 0.015), however risk estimates for both strata included one. For gastric cardia adenocarcinoma, the risk estimate for never smokers was significant, but the interaction test was not (P = 0.33), suggesting no difference in effect from the overall null estimate.

Table 3 presents the associations between use of nine individual vitamin or mineral supplements and upper gastrointestinal cancer risk. ESCC showed no significant associations. Esophageal adenocarcinoma risk showed an inverse association with iron supplement use (HR = 0.68, 95% CI = 0.49 to 0.94). Risk of both esophageal adenocarcinoma and gastric cardia adenocarcinoma, showed direct associations with calcium supplement use (HR = 1.27, 95% CI = 1.06 to 1.52) and (HR = 1.27, 95% CI = 1.03 to 1.56), respectively. Gastric noncardia adenocarcinoma risk showed a direct association with iron (HR = 1.59, 95% CI = 1.24 to 2.05) and zinc supplement use (HR = 1.28, 95% CI = 1.01 to 1.62), but an inverse association with vitamin C supplement use (HR = 0.79 95% CI = 0.65 to 0.96) (Table 3).

**Table 3:** Adjusted* hazard ratios (HR) and 95% confidence intervals (CI) for use of individual vitamin or mineral supplements for four upper gastrointestinal cancers in the NIH-AARP Diet and Health Study cohort.

	Supplement users N (%)	ESCC	EADC	GCA	GNCA
		None, N (%)/Any**, N (%)	None, N (%)/Any, N (%)	None, N (%)/Any, N (%)	None, N (%)/Any, N (%)
		HR (95% CI)	HR (95% CI)	HR (95% CI)	HR (95% CI)
**Iron**	50193 (10)	190 (90)/22 (10)	586 (94)/39 (6)	405 (90)/45 (10)	422 (86)/71 (14)
		1.13 (0.72–1.76)	0.68 (0.49–0.94)	1.12 (0.82–1.52)	1.59 (1.24–2.05)
**Zinc**	66039 (13)	190 (90)/22 (10)	553 (88)/72 (12)	394 (88)/56 (12)	412 (84)/81 (16)
		0.82 (0.52–1.28)	0.85 (0.67–1.09)	0.94 (0.71–1.25)	1.28 (1.01–1.63)
**Selenium**	38064 (8)	200 (94)/12 (6)	580 (93)/45 (7)	422 (94)/28 (6)	449 (91)/44 (9)
		0.81 (0.45–1.45)	1.02 (0.75–1.38)	0.87 (0.59–1.28)	1.25 (0.91–1.70)
**Calcium**	170892 (35)	150 (71)/62 (29)	451 (72)/174 (28)	319 (71)/131 (29)	349 (71)/144 (29)
		0.92 (0.67–1.25)	1.27 (1.06–1.52)	1.27 (1.03–1.56)	0.94 (0.77–1.16)
**Folic Acid**	42999 (9)	194 (92)/18 (8)	582 (93)/43 (7)	413 (92)/37 (8)	447 (91)/46 (9)
		1.05 (0.65–1.71)	0.82 (0.60–1.13)	1.00 (0.72–1.41)	1.14 (0.84–1.54)
**Vitamin A**	67018 (14)	186 (88)/26 (12)	552 (88)/73 (12)	396 (88)/54 (12)	414 (84)/79 (16)
		0.97 (0.64–1.47)	0.91 (0.71–1.16)	0.94 (0.70–1.25)	1.27 (1.00–1.61)
**β-carotene**	79706 (16)	185 (87)/27 (13)	536 (86)/89 (14)	389 (86)/61 (14)	414 (84)/79 (16)
		0.84 (0.56–1.26)	0.96 (0.76–1.20)	0.91 (0.69–1.19)	1.06 (0.83–1.36)
**Vitamin C**	205112 (42)	139 (66)/73 (34)	397 (64)/228 (36)	270 (60)/180 (40)	324 (66)/169 (34)
		0.81 (0.60–1.08)	0.93 (0.79–1.10)	1.09 (0.90–1.32)	0.79 (0.65–0.96)
**Vitamin E**	191352 (39)	145 (68)/67 (32)	406 (65)/219 (35)	282 (63)/168 (37)	322 (65)/171 (35)
		0.79 (0.59–1.06)	0.95 (0.81–1.13)	1.06 (0.87–1.28)	0.87 (0.72–1.06)

*Adjustments included age at cohort entry, sex, education, smoking status and intensity, alcohol use, fruit intake, vegetable intake, body mass index (BMI), vigorous physical activity, usual physical activity during the day, and total energy intake.

**Any use defined as reporting use more than once per month. ESCC = esophageal squamous cell carcinoma; EADC = esophageal adenocarcinoma; GCA = gastric cardia adenocarcinoma; GNCA = gastric noncardia adenocarcinoma.

## Discussion

We examined the association of multivitamin and single vitamin or mineral supplement use with risk for cancers of the esophagus or stomach in a large US prospective cohort where vitamin supplement use is common. Multivitamin use had no significant main effect associations with risk of ESCC, esophageal adenocarcinoma, gastric cardia adenocarcinoma, or gastric noncardia adenocarcinoma cancer. We tested for interactions by sex or smoking status and found one significant difference for esophageal adenocarcinoma risk by smoking status, but risk estimates for both strata included unity and it seems unlikely that this reflects a real difference in effect. Previous studies, both observational and interventional, have reported that multivitamin supplements have no association with risk of upper gastrointestinal cancers [12]–[13], even in populations with poor nutritional status [18]. We recently published extended follow-up from a multivitamin supplementation trial in a nutritionally deficient Chinese population that showed that even in this setting, multivitamins had no preventive effect on upper GI cancer risk [19]. Our results are consistent with these studies.

For the individual supplements, use of iron supplements was associated with significantly lower risk of esophageal adenocarcinoma and a significantly higher risk of gastric noncardia adenocarcinoma. For gastric noncardia adenocarcinoma, either iron or zinc supplement use was associated with increased risk, whereas vitamin C use was associated with lower risk. Calcium use significantly increased the risk for esophageal adenocarcinoma and gastric cardia adenocarcinoma cancers.

We found that use of iron supplements was associated with significantly lower risk of esophageal adenocarcinoma and a significantly increased risk of gastric noncardia adenocarcinoma. In animal models with surgically induced reflux, high doses of iron can induce esophageal adenocarcinomas [20]. In almost all populations, regardless of the incidence rates, upper gastrointestinal cancers are male predominant. Higher iron status, which is characteristic of men, has been hypothesized as one explanation for this phenomenon. But few studies have examined iron supplements and risk of any upper gastrointestinal cancer. We note that a several recent studiesy showed that subjects with higher dietary iron intake of non-heme iron or high iron stores had lower risk of Barrett’s esophagus, the preneoplastic lesion for esophageal adenocarcinoma [21] and esophageal adenocarcinoma [22].

Vitamin C supplementation and cancer have been widely investigated, but only a limited number have examined vitamin C supplements and upper GI cancers. A hospital-based case-control study, the Cancer Prevention Study II cohort, and a study from Seattle’s Barrett’s Esophagus Program showed protective effects of vitamin C supplementation on esophageal cancer [23], gastric cancer [11], and esophageal adenocarcinoma [24]. Serum vitamin C deficiency may increase risk of gastric cancer [25]. In the current study, use of vitamin C as a single supplement was associated with lower risk of gastric noncardia cancer, but there was no effect of multivitamin supplements, which usually contain vitamin C. This difference may be due to the generally lower dose in multivitamin supplements or to antagonism from other multivitamin components.

We found a higher risk of esophageal adenocarcinoma and gastric cardia adenocarcinoma among those using calcium supplements. In our questionnaire, we cannot separate the use of calcium containing antacids as a calcium supplement from the use of these to treat heartburn/gastroesophageal reflux disease. Reflux disease is a primary risk factor for adenocarcinomas at the gastroesophageal junction [26]–[29] and this likely explains these findings.

Higher selenium status has been associated with lower risk of ESCC and gastric cardia adenocarcinoma in previous studies [30]–[31] and in a randomized controlled trial a supplement containing selenium reduced the risk of ESCC and gastric cancer in the NIT General Population Trial [6]. We found no associations between selenium supplement use and any of the four cancer sites here. This difference in results may be due to the generally higher selenium status in the US, whereas some other populations have lower status. The effect of selenium supplements on cancer risk may be dependent on the underlying selenium status of the population [32].

Our study had several strengths, including the large size and prospective nature, which provide good power and minimizes recall bias. We also had the ability to control for numerous potential confounders. The importance of this is evident in table 2 when comparing the crude and adjusted models. Limitations of the study include not having collected lifetime supplement use and having insufficient dose information and sufficient number of incident cancers for most supplements to accurately estimate associations by model dose for individual supplements. We also lacked information on some upper Gi cancer risk factors that could confound our estimates including GERD, Barrett’s esophagus and peptic ulcer information on all subjects at baseline. Our results may not be generalizable to other populations with different demographic characteristics, underlying cancer risks, or nutritional status.

In summary, we found no evidence that multivitamin use was associated with reduced risk of four highly fatal upper gastrointestinal cancers, but there were some differences in risk with reported use of individual supplements. These latter findings require confirmation in other prospective studies.
